# Highly sensitive and portable mRNA detection platform for early cancer detection

**DOI:** 10.1186/s12951-021-01039-4

**Published:** 2021-09-26

**Authors:** Hongxia Li, Antony R. Warden, Wenqiong Su, Jie He, Xiao Zhi, Kan Wang, Laikuan Zhu, Guangxia Shen, Xianting Ding

**Affiliations:** 1 State Key Laboratory of Oncogenes and Related Genes, Institute for Personalized Medicine, School of Biomedical Engineering, Shanghai Jiao Tong University, Shanghai, 200030 China; 2 Institute of Nano Biomedicine and Engineering, Shanghai Engineering Research Centre for Intelligent Diagnosis and Treatment Instrument, Department of Instrument Science and Engineering, School of Electronic Information and Electrical Engineering, Shanghai Jiao Tong University, Shanghai, 200240 China; 3 Department of Endodontics and Operative Dentistry, Ninth People’s Hospital, Shanghai Jiao Tong University School of Medicine, Shanghai, 200011 China; 4 Shanghai Key Laboratory of Stomatology & Shanghai Research Institute of Stomatology, National Clinical Research Center of Stomatology, Shanghai, 200030 China

**Keywords:** Early-stage cancer detection, mRNA, Lateral flow assay, Point-of-care testing, Glypican-1

## Abstract

Pancreatic cancer, at unresectable advanced stages, presents poor prognoses, which could be prevented by early pancreatic cancer diagnosis methods. Recently, a promising early-stage pancreatic cancer biomarker, extracellular vesicles (EVs) related glypican-1 (GPC1) mRNA, is found to overexpress in pancreatic cancer cells. Current mRNA detection methods usually require expensive machinery, strict preservation environments, and time-consuming processes to guarantee detection sensitivity, specificity, and stability. Herein, we propose a novel two-step amplification method (CHAGE) via the target triggered Catalytic Hairpin Assembly strategy combined with Gold-Enhanced point-of-care-testing (POCT) technology for sensitive visual detection of pancreatic cancer biomarker. First, utilizing the catalyzed hairpin DNA circuit, low expression of the GPC1 mRNA was changed into amplification product 1 (AP1, a DNA duplex) as the next detection targets of the paper strips. Second, the AP1 was loaded onto a lateral flow assay and captured with the gold signal nanoparticles to visualize results. Finally, the detected results can be further enhanced by depositing gold to re-enlarge the sizes of gold nanoparticles in detection zones. As a result, the CHAGE methodology lowers the detection limit of mRNA to 100 fM and provides results within 2 h at 37 °C. Furthermore, we demonstrate the successful application in discriminating pancreatic cancer cells by analyzing EVs’ GPC1 mRNA expression levels. Hence, the CHAGE methodology proposed here provides a rapid and convenient POCT platform for sensitive detection of mRNAs through unique probes designs (COVID, HPV, etc.).

**Supplementary Information:**

The online version contains supplementary material available at 10.1186/s12951-021-01039-4.

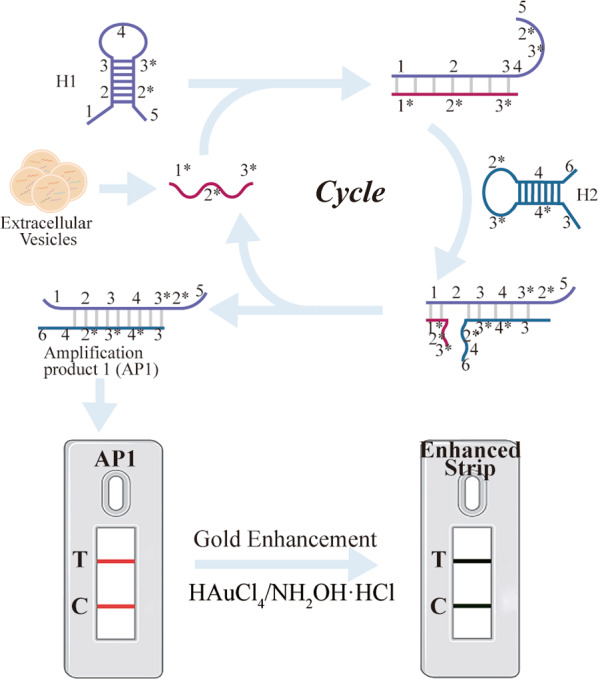

## Introduction

Pancreatic cancer, one of the most lethal cancers, has poor prognosis, hard diagnosis, and rapid progression, with only 4% of patients that survive 5 years after diagnosis [1, 2]. Notably, due to poor early detection of pancreatic cancer, patients diagnosed are usually in the advanced stage and have a median survival time of 3–14 months [3, 4]. Therefore, highly specific detection of pancreatic cancer at its early stages is critical to effectively intervention and treatment. In recent years, extracellular vesicles (EVs) with high glypican-1 (GPC1) expression level have been regarded as effective potential biomarkers for pancreatic cancer diagnosis [5]. The amount of EVs related GPC1 mRNA expression level could relate to the periods of pancreatic cancer. Hence, sensitive detection of EVs’ GPC1 mRNA expression would be an effective approach in early-stage pancreatic cancer diagnosis [1–4].

Sensitive mRNA identification is challenging because of its inherent characteristics, such as low expression levels and sequence similarities [6–8]. Current methods that offer accurate and sensitive mRNA analysis, e.g., northern blotting and quantitative reverse transcriptase-polymerase chain reaction (qRT-PCR), are laborious, time-consuming, and expensive [9, 10]. Other prevailing mRNA detection methods, such as rolling circle amplification (RCA) and loop-mediated isothermal amplification (LAMP) [11–13] are limited by their requirement of expensive protein enzymes, strict preservation environments, and precise experimental conditions [6, 14]. Hence, it is critically important to develop a platform that provide sensitive, stable, and portable detection of mRNA.

Recently, point-of-care-testing (POCT) technology combined with nanomaterials shows remarkable diagnosis results in resource-limited environments while being portable [15–17]. Paper-based platforms are becoming one of the most popular POCT methods owing to their low-cost, direct result readout, and long-term storage viability [18–21]. Paper-based strips can accurately detect protein, miRNA, and viruses [19, 22–24]. The addition of functional nanomaterials, such as colloidal gold nanoparticles (AuNPs), quantum dots (QDs), and magnetic beads, to paper-based strips, can further improve detection precision [18, 25].

To provide a sensitive, stable, and portable mRNA detection platform, we developed a two-step mRNA signal amplification strategy with femtomolar resolution based on Catalytic Hairpin Assembly (CHA) and Gold-Enhanced strips (CHAGE strips) [26–28]. First, target-triggered hairpin assembly is prepared for preliminary mRNA amplification. Next, the amplified product is trapped in the test zone on the strips with gold signal probes. Then, in situ gold deposition via HAuCl_4_/NH_2_OH·HCl reaction was applied to amplify the detection sensitivity of the CHAGE strips. CHAGE strips provide a femtomolar mRNA detection platform within 2 h. At last, we used EVs related GPC1 mRNA as a target, and this platform successfully identified the pancreatic cancer cell line (AsPC-1) [1]. This method provides a potential strategy for sensitive mRNA detection which is beneficial to early-stage diagnosis or prognosis of pancreatic cancer. In summary, this study provides a rapid, convenient, and sensitive mRNA detection platform, which could be adopted for detecting all sources of mRNA (e.g., COVID-19, HPV) through relevant probes design.

## Materials and methods

### Materials

The Airjet AJQ 3000 dispenser, Biojet BJQ 3000 dispenser, and Guillotine cutting module CM 4000 were purchased from BioDot (CA, USA). The HP LaserJet Professional M1216nfh MFP is purchased from the Hewlett-Packard Company (CA, USA). The ultracentrifuge (OPTIMA XPN-100) is purchased from Beckman (CA, USA). Gold (III) chloride trihydrate (HAuCl_4_·3H_2_O), sodium citrate tribasic dehydrate (Na_3_C_6_H_5_O_7_·2H_2_O), streptavidin, bovine serum albumin (BSA), and tris (2-carboxyethyl) phosphine hydrochloride solution (TCEP) were purchased from Sigma-Aldrich (MO, USA). All oligonucleotides (Additional file 1: Table S1) were synthesized by Sangon Biotech (Shanghai, China). Tween-20, sodium chloride-sodium citrate buffer (SSC, 20×, pH = 7.0), phosphate-buffered saline (PBS, 1×, pH = 7.4), and Triton X-100 were purchased from Sangon Biotech (Shanghai, China). Cellulose fiber sample pad (JY-Y107), conjugate pad (JY-BX101), nitrocellulose membrane (NC membrane) (Millipore 135), absorbent pad, and plastic adhesive baseboard (H5015) were purchased from Jieyi Biotechnology (Shanghai, China). Sucrose, hydroxylamine hydrochloride (NH_2_OH·HCl), and sodium chloride (NaCl) were purchased from Sinopharm Chemical Reagent (Shanghai, China). CD63 was purchased from NOVUS (CO, USA). TSG101 was purchased from Santa Cruz (Texas, USA). Calnexin was purchased from CST (MA, USA). All reagents were used without further purification.

### Au nanoparticle synthesis

AuNPs was prepared according to the citrate reduction method with minor modification [29]. Before use, all glassware was soaked in aqua regia (HCl/HNO_3_:3/1) and cleaned. 100 mL of 0.01% (w/w) HAuCl_4_ solution was boiled with vigorous stirring, then 1.5 mL of 1% (w/w) trisodium citrate aqueous solution was rapidly added to the boiling solution. Within minutes, the solution color changed from light yellow to vinaceous red. The reaction solution was boiled for 30 more minutes to guarantee the complete reduction of the gold nanoparticles. The colloidal AuNPs suspension was cooled to room temperature while being continuous stirred. The desired gold colloidal solution was stored in brown glass at 4 °C.

### Hairpin probe design

The design of the hairpin probes (listed 5′–3′) was based on a non-enzyme amplification strategy. The target sequence designed was based on NCBI’s reference sequence of GPC1 mRNA (NM-002081.2 location 2034). The sequence of location 2034 is CTC TGA GCA GGG GCA GGC. The sequence (listed 5′–3′) of hairpin 1 (H1) designed for location 2034 is: GCC TGC CCC TGC TCA GAG AGA ATG TGA ACA CTC TGA GCA GGC CTT GTC ATA GA. The sequence (listed 5′–3′) of hairpin 2 (H2) designed for location 2034 is: CAG AGT GTT CAC ATT CTC TCT GAG CAT AAG AAT GTG AAC AGA CAC CAT TT. These two hairpin probes (H1 & H2) were heated to 95 °C for 5 min and cooled to room temperature within 2 h to ensure their folding into a hairpin structure. All reagents were prepared in PBS buffer (1×). The probe solution was stored in 4 °C.

### Gold signal probe preparation

The DNA probe design of gold signal probe was based on hairpin probes. The DNA probe sequence (listed 5′–3′) is (5′SH-C6) TCT ATG ACA AGG. The DNA probe was modified with thiol to produce the Au signal probe, following previous literature with little modification [30]. Briefly, 1 OD 5′-thiol modified signal probes were added into freshly prepared AuNPs (1 mL) at tenfold concentration. The solution was gently shaken overnight. Subsequently, 1 M NaCl was added into the mixture at a low rate until a final concentration of 0.1 M NaCl was achieved. It was then incubated with 1% BSA to passivate the Au signal probes for 30 min at 37 °C. Excess reagents were removed via centrifugation at 10,000 rpm for 30 min. Then, the wash steps were repeated three times to ensure the complete removal of excess reagents. The resulting Au signal probes were stored in 20 mM Na_3_PO_4_·12H_2_O, 5% BSA, 0.25% Tween-20, and 10% sucrose at 4 °C for further use.

### Streptavidin-biotinylated DNA conjugate preparation

The DNA probe design of control zone and test zone was based on hairpin probes. The control zone DNA probe sequence (listed 5′–3′) is (5′Biotin) CCT TGT CAT AGA. The test zone DNA probe sequence (listed 5′–3′) is (5′Biotin) AAA TGG TGT C. The control zone probe and test zone probe were synthesized based on the streptavidin-biotin system. 200 µL of streptavidin at 2.5 mg/mL and 50 nmol biotinylated DNA probe (C line probe/T line probe) were mixed and stirred at 37 °C for 1 h. Then, 500 µL PBS (1×) was added to the mixture. Next, the solution was centrifuged with an ultra-filtration tube for 20 min at 6000 rpm under 4 °C. The above procedures were repeated 3 times to ensure complete removal of unbound DNA. The remaining solution was diluted to 600 µL with PBS (1×) and stored at 4 °C.

### Biosensor strip assembly

The biosensor strip was made up of four components: a sample pad, a conjugation pad, a nitrocellulose (NC) membrane, and an absorbent pad. All components were assembled onto a plastic adhesive baseboard and the pads overlapped with each other for 2 mm to guarantee that the solution would smoothly migrate through the strip. The sample pad was soaked in 3% BSA, 0.1 M NaCl, 1% Triton X-100 in 0.1 M Tris-HCl (pH = 8.0) buffer, while the conjugated pad was immersed in 4% sucrose and 1% Tween 20 in 0.1 M Tris-HCl (pH = 8.0) buffer. After 30 min, these two pads were dried at 37 °C for 1 h. The test and control lines on the nitrocellulose membrane were dispensed using streptavidin-biotinylated probes solution. Additionally, the NC membrane was dried at 37 °C for 1 h. Last, the integrated plate was cut into a width of 3 mm and stored in a desiccator for later use.

### Assay procedure

Typical mRNA detection entails two amplification reactions on the strips. The first amplification system operates at 37 °C for 90 min with a total volume of 50 µL. It results in different concentrations of H1, H2, and mRNA in the PBS (1×). At the end of the reaction, the product of the first amplification step was loaded onto the sample pad and the solution migrated with capillary force. After reacting with the signal probe on the conjugate pad, the control line and test lines appeared within 6 min. Then, 50 µL washing buffer was applied onto the sample pad to wash the unconjugated signal on the NC membrane. After 10 min, a 1.5 µL signal amplification solution was independently added onto the control and test lines. The AuNPs, as an Au seed, would grow larger with the reduction of HAuCl_4_, producing a darker color that enhances signal intensity. The entire amplification process occurred within 2 h.

### Data analysis of strips

For quantitative measurements of target concentration, the photographs of strips were recorded with a scanner. Then we adjusted the color strips photograph to grayscale images, and the peak area of the test line was analyzed using Image J software.

### Cell culture and EVs isolation

Human pancreatic carcinoma cell line (AsPC-1) and Human Pancreatic Nestin Expressing cells (HPNE) were cultured at 37 °C with 95% air and 5% CO_2_ in an incubator and maintained in an RPMI-1640 medium with 10% FBS and 1% penicillin-streptomycin. The isolation of EVs followed previous literature [5]. The medium was centrifuged at 3000 rpm for 10 min to remove cells and cellular detritus. Next, the medium was centrifuged at 10000×*g* for 10 min to remove large vesicles, then the medium was filtered using a 0.22 μm pore filter. The filtered medium was ultra-centrifuged at 100000×*g* for 90 min at 4 °C to collect EVs, then the pellet was suspended in PBS (1×) and centrifuged at 100000×*g* for 90 min at 4 °C. The pellet was re-suspended in 200 µL sterile PBS (1×) and centrifuged at 3000×*g* for 10 min to remove EVs aggregates formed during ultra-centrifugation. The collected EVs solution was used for transmission electron microscopy, nanoparticle tracking analysis, western blot, and RNA extraction.

### RNA extraction from EVs

The RNA extraction was conducted following the manufacturer’s protocol. The RNA of the EVs was extracted using the TRIzol Plus RNA Purification kit (Thermo Fisher Scientific, USA). Briefly, 1 mL TRIzol solution was added into collected EVs solution and incubated for 5 min. Then 200 µL chloroform was added into the tube and incubated for 5 min. The complex solution was centrifuged at 12000×*g* for 15 min at 4 °C. Transferring colorless solution into a new tube and added equal volume of 75% ethanol then mixed well. The complex solution was centrifuged at 12000×*g* for 5 min at 4 °C, The pellets were collected and suspended in 30 µL DEPC water for further use.

## Results and discussions

### Principle of amplification

**Fig. 1:**
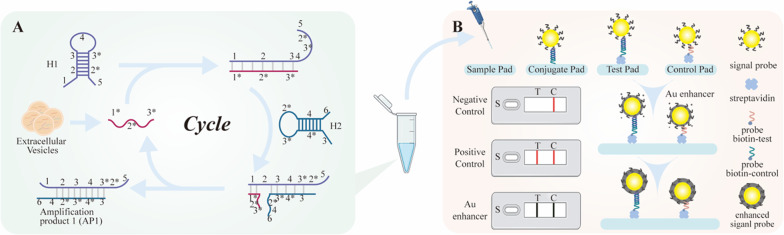
The principle of the CHAGE strip for mRNA detection. **A** In the first amplification step, H1 and H2 hairpins are designed based on the target. When the target exists, it will react with H1, enabling reaction with H2, which releases the target for further amplification. **B** In the second amplification step, the product from the first amplification step reacts with gold signal particles, and then trapped in the testing zone, with the control zone guaranteeing the strip’s validity. Lastly, the Au enhancer buffer (HAuCl_4_/NH_2_OH·HCl) improves the detection sensitivity via gold deposition enhancement. The strips zone illustrates positive and negative conditions

The overview of the proposed CHAGE strips strategy for sensitive mRNA detection is depicted in Fig. 1. The procedure contains mRNA signal amplification and paper-based strip detection combined with Au enhanced signal for sensitive detection of mRNA. The mRNA signal amplification utilizes a CHA procedure (Fig. 1A). The research proved the CHA method is an effective and specific mRNA amplification strategy [5, 31, 32]. Here, the GPC1 mRNA 2034 region is selected as a proof-of-concept target. Hairpin 1 (H1) and hairpin 2 (H2) are designed based on the GPC1 mRNA sequence, which can coexist as folded structures. In the presence of the GPC1 mRNA, the toehold of H1 reacts with it to form an H1-mRNA complex. As a result, the unfolded H1 will present a new single-strand region that reacts with the toehold of H2, releasing the GPC1 mRNA for a further round of amplification. The amplification product 1 (AP1) is loaded onto a lateral flow assay (LFA) platform for visual observation (Fig. 1B). AP1 reacts with the gold signal particles on the conjugate pad and is trapped in the testing zone. When mRNA is absent, the signal probes are only trapped in the control zone for visualization. In the second amplification step, HAuCl_4_ is deposited around AuNPs which result in darker color to increase signal intensity.

In summary, the CHA process amplifies unstable long-sequence mRNA to stable short-sequence products (AP1). The enhanced LFAs signal readout method is sensitive, convenient, and economical while also permits visual signal readout without specific machines. The enhance process in LFAs generates gold deposits around AuNPs to widen test zone grayscale by in situ reduction HAuCl_4_. As a result, this CHAGE strips strategy offers a novel platform for sensitive mRNA detection, with results observable with the naked eye. Quantitative analysis can be performed with visual color devices, such as scanners or smartphones.

### Amplification feasibility analysis

As shown in Fig. 1A, we separate the target mRNA sequence-specific region into 3 parts: 1*, 2*, and 3*. The stem-loop DNA hairpin 1 (H1) contains seven parts: 1, 2, 3, 4, 3*, 2*, and 5; H2 has 6 parts: 3, 4*, 3*, 2*, 4, and 6. When the target mRNA is present, it reacts with the H1 toehold fragment 1, which unfolds H1 and forms an H1-mRNA complex, the new naked 3* fragment in H1 will then react with the toehold of H2. The stability of the H1–H2 complex will release the target mRNA, allowing it to react with additional H1 toeholds.

**Fig. 2:**
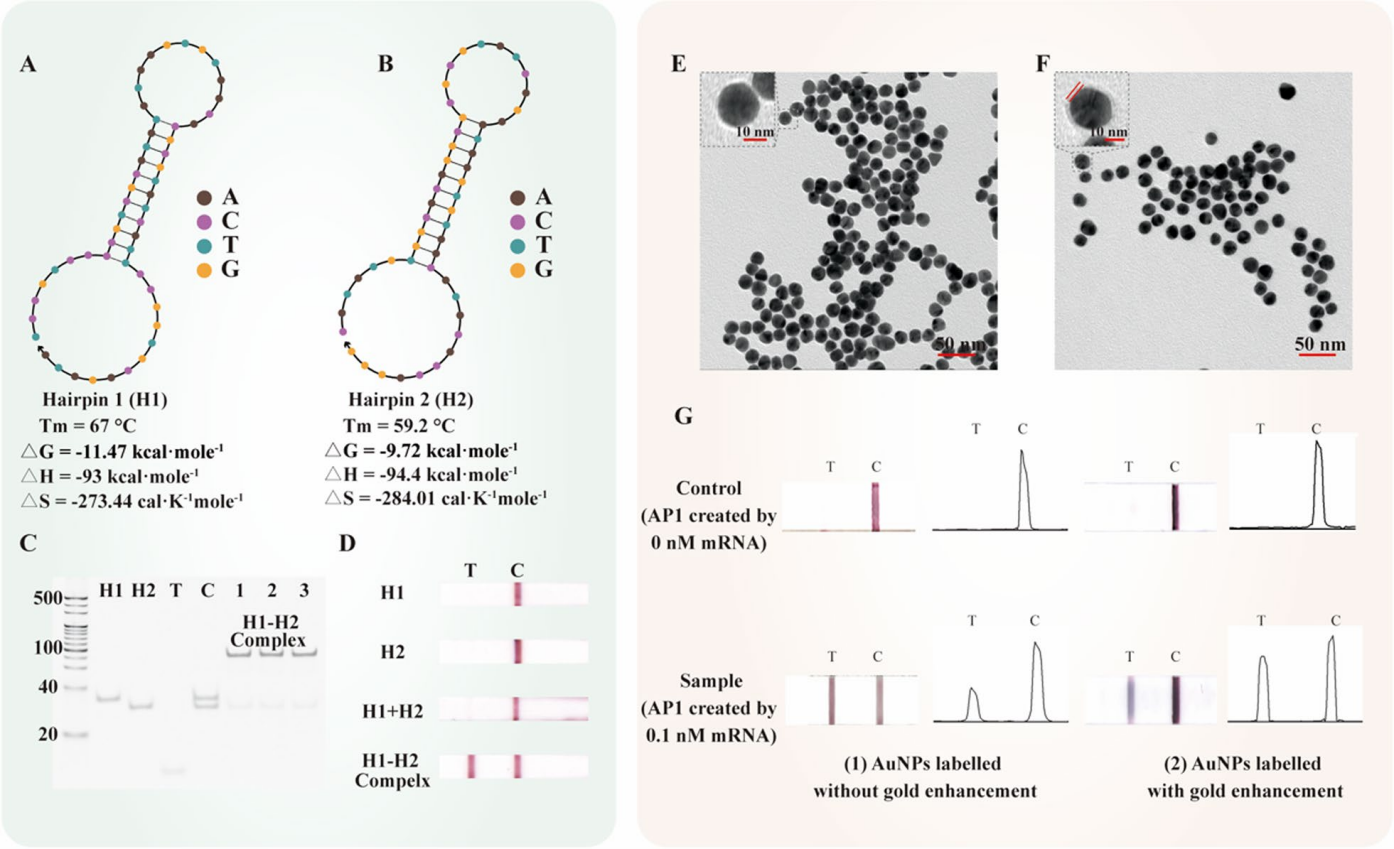
Validation of the amplification steps. Simulation of the structure and thermodynamics parameters of H1 (**A**) and H2 (**B**). **C** 12% PAGE of the product of the first amplification step. Ladder: 20 bp DNA marker, H1: hairpin 1, H2: hairpin 2, T: mRNA target, C: negative control, lanes 1–3: 3 separate products of the first amplification step. **D** LFAs data of all components in first amplification step [H1, H2, H1 + H2(Control) and H1 + H2 + T(Sample)]. **E** TEM of gold nanoparticles. **F** TEM of gold signal probes. **G** Photographs and corresponding grayscale map of the second amplification step, (1): AuNPs labelled strips of AP1 (created by 0 nM and 0.1 nM mRNA) without gold enhancement; (2): AuNPs labelled strips of AP1 (created by 0 nM and 0.1 nM mRNA) with gold enhancement. [Amplification step reagents: 0.1% (w/w) HAuCl_4_ and 10 mM NH_2_OH·HCl (1:10), reaction time: 5 min]

We use Oligoanalyzer 3.1™ and NUPACK™ to analyze probe feasibility. As Fig. 2A, B outlines, the high melting temperatures of H1 and H2 guarantee the stability of the stem-loop hairpin structure. The low Gibbs free energy of the reaction product ensured the efficiency of the reaction and stable formation of the H1–H2 complex. Other relevant parameters of H1 and H2 are also provided in Fig. 2A, B (simulation in Additional file 1: Figure S1). To further investigate probe feasibility, the amplification products are qualified with 12% polyacrylamide gel electrophoresis (PAGE) with the coloration of Gel-red. The detailed conditions are shown in Fig. 2C. The control lane indicates that H1 and H2 do not spontaneously react at the reaction temperature. Strips 1–3 are three parallel amplification experiments of 10 nM target mRNA zones. To guarantee completed reaction, the proportion of H1 to H2 is 1:1.5, which would result in some leftover H2 left after the reaction. Strong bands indicate most product was H1–H2 complex. In Fig. 2D, we verified the effectiveness of using strips to readout the first-amplification step results. In the conditions of absent target (H1, H2, H1 + H2), there was no gold signal probes aggregated on the test zones of strips. Only the amplification product 1 (H1–H2 complex) could cause positive signal in test zones of strips. In summary, PAGE and LFA results demonstrate the specific amplification product of H1–H2 complex, confirming the validity of the first amplification step.

Next, TEM images (Fig. 2E, F) show the successfully binding of nucleic acid probe on gold nanoparticles. Furthermore, the stability of gold signal probes in high salt buffer (Additional file 1: Figure S2) and the new wavelength peak of 260 nm in gold signal probes UV–Vis’s measurement (Additional file 1: Figure S3) revalidate the successfully binding of nucleic acid probe on gold nanoparticles. The photographs of the strips confirm that the signal probe has a low background signal and great stability in CHA product detection (Fig. 2G). Furthermore, increase of grayscale and visual effect on T lines under enhancer buffer treatment was significant. In addition, no visible band was observed on the T lines in the control sample. The UV–Vis of signal probes before and after amplification (Additional file 1: Figure S4) indicates that the diameter of signal probe increased after amplification (a red shift of character peak). Therefore, the second amplification step based on AuNP is suitable in combination with the first amplification (CHA) for sensitive and portable mRNA detection.

### Experimental condition optimization

**Fig. 3:**
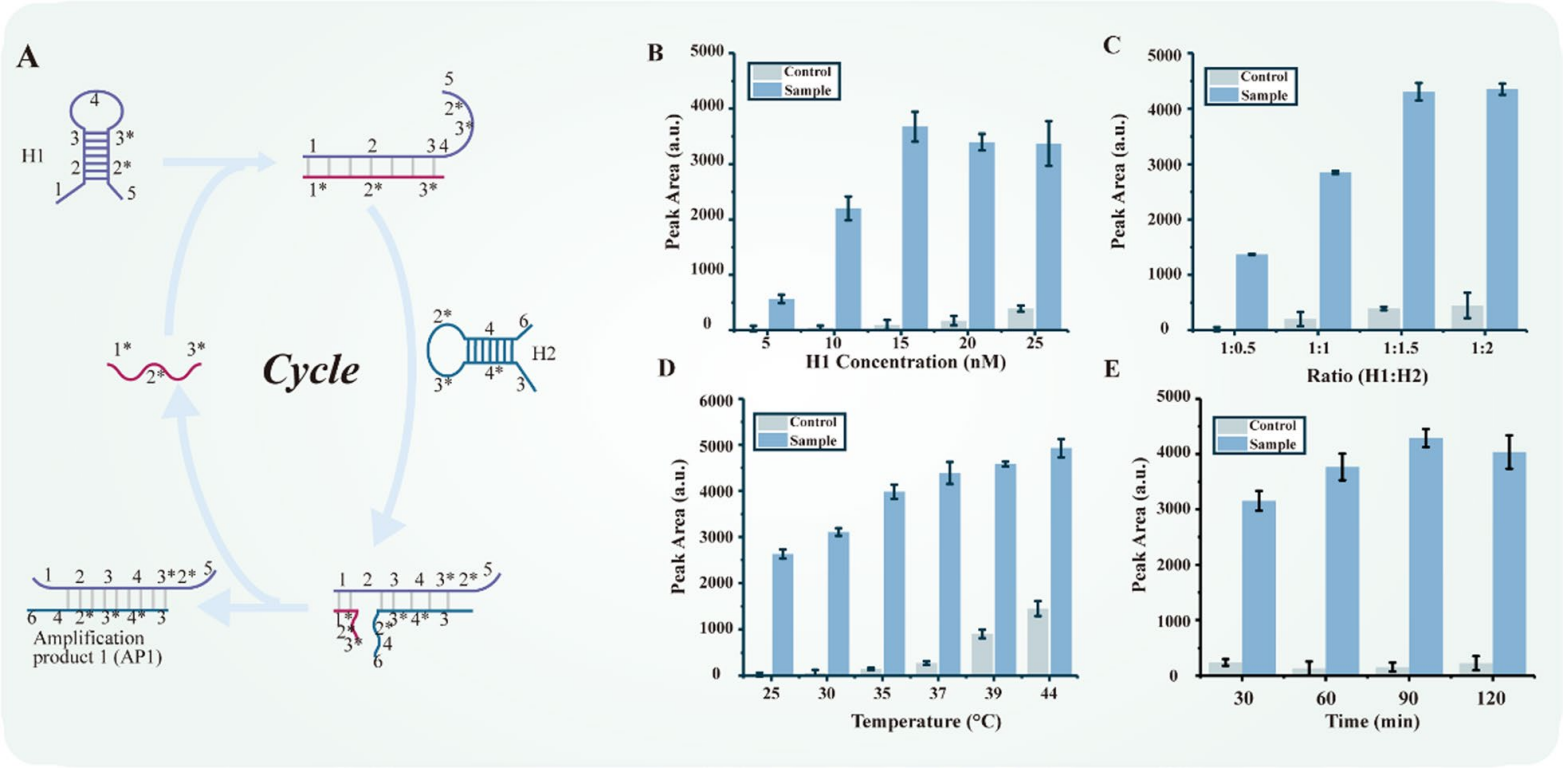
Optimization of experimental parameters of CHA process. **A** The principle of the CHA process. **B** Varying concentrations of initial H1 and H2 (5 nM, 10 nM, 15 nM, 20 nM, and 25 nM) with fixed ratio at 1:1. **C** Varying ratios between H1 and H2: 1:0.5, 1:1, 1:1.5, and 1:2, with fixed H1 concentration at 15 nM. **D** Varying incubation temperatures from 25 to 44 °C with optimized concentration and ratio. **E** Varying incubation time length for CHA amplification (30–120 min) with optimized conditions. All error bars represent mean ± s.d., n = 3

We optimize the experimental setups for enhanced sensitivity and reproducibility. The initial concentrations of H1 and H2 are important for the detection of target mRNA in the CHA process (Fig. 3). Specifically, 1 nM target mRNA was examined with different concentrations of H1. Five different concentrations of H1 (5, 10, 15, 20, and 25 nM) are tested with the same concentration of H2. The signal intensity saturated after 15 nM and the signal at the control zones gradually increased after 15 nM (Fig. 3B). Hence, we determine 15 nM as the optimal reaction concentration.

The ratio of H1 to H2 is vital to enhancing the H1–H2 complex formation efficiency. Here, we tested four different ratios of H1 to H2. As illustrated in Fig. 3C, ratios of 1:0.5, 1:1, 1:1.5, and 1:2 are tested, with H1 concentration fixed at 15 nM. As excess of H2 would accelerate complex formation, the signal intensity increased with the addition of H2. When the ratio is 1:1.5, the signal of the test sample is the strongest and the signal of the control sample was acceptable. Therefore, we determine that the optimal condition as 15 nM H1, with H1 to H2 ratio as 1:1.5.

To further optimize CHA experiment parameters, the incubation temperature is examined (Fig. 3D). Temperatures between 25 and 44 °C are tested to evaluate its influence in CHA process. As the temperature rises, the signal and background noise intensities also increase. At 37 °C, the signal intensity is almost as strong as higher temperatures, while the background signal was tolerable compared to higher temperatures. Therefore, 37 °C is determined as the optimal experiment temperature in CHA process.

To obtain optimal amplification and economic efficiency, the reaction time of CHA process is examined. As shown in Fig. 3E, at varying incubation time lengths, signal intensity reached a plateau at 90 min. Therefore, 90 min is considered as the optimal CHA reaction time.

**Fig. 4:**
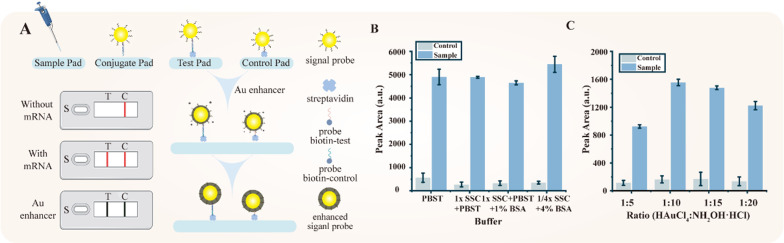
Optimization of experimental parameters of the LFA process. **A** The principle of signal enhanced LFAs. **B** Different washing buffers of strips for cleaning unconjugated signal probe: PBST, SSC (1×) + PBST, SSC (1×) + PBST + 1 %BSA, SSC (1/4×) + 4% BSA. **C** Varying volume ratios between 1% HAuCl_4_ and 10 mM NH_2_OH·HCl: 1:5, 1:10, 1:15, 1:20. All error bars represent mean ± s.d., n = 3

As for the second amplification step (Fig. 4), the washing buffer is considered as the most critical factor that influences the target and background signal intensity. Hence, we compared the following four types of buffers: buffer 1: PBST (PBS (1×, pH = 7.4) with 0.5% Tween-20), buffer 2: SSC (1×) with PBST, buffer 3: SSC (1×) with PBST and 1% BSA, and buffer 4: SSC (1/4×) with 4% BSA. The results of the buffers are shown in Fig. 4B, specifically, the test line in the buffer (SSC (1/4×) with 4% BSA) markedly increased, presumably because the SSC (1/4×) buffer positively influences nucleic acid hybridization and 4% BSA efficiently reduces non-specificity combination. Therefore, 1/4× SSC combined 4 %BSA is determined as the optimal running buffer.

The proportion of HAuCl_4_ and NH_2_OH·HCl is a significant condition for the second amplification step. We first validate the enhanced ability in the solution (Additional file 1: Figures S5–S7). The grayscale and wavelength of four ratios [1% HAuCl_4_ and 10 mM NH_2_OH·HCl (1:5, 1:10, 1:15 and 1:20)] are stabled in 10 min. when these solution parameters were applied in the strips, the target concentration was fixed at 10 pM with a ratio of 1:10 (1% HAuCl_4_ and 10 mM NH_2_OH·HCl) that had the strongest signal for the test sample with acceptable control sample intensity (Fig. 4C). In a certain volume put on strips, the amount of NH_2_OH·HCl reduced the proportion of HAuCl_4_ will decrease the reaction rate. Therefore, 1:10 ratio of 1% HAuCl_4_ and 10 mM NH_2_OH·HCl is determined as the optimal ratio.

### Sensitivity and specificity of the CHAGE strips for mRNA detection

**Fig. 5:**
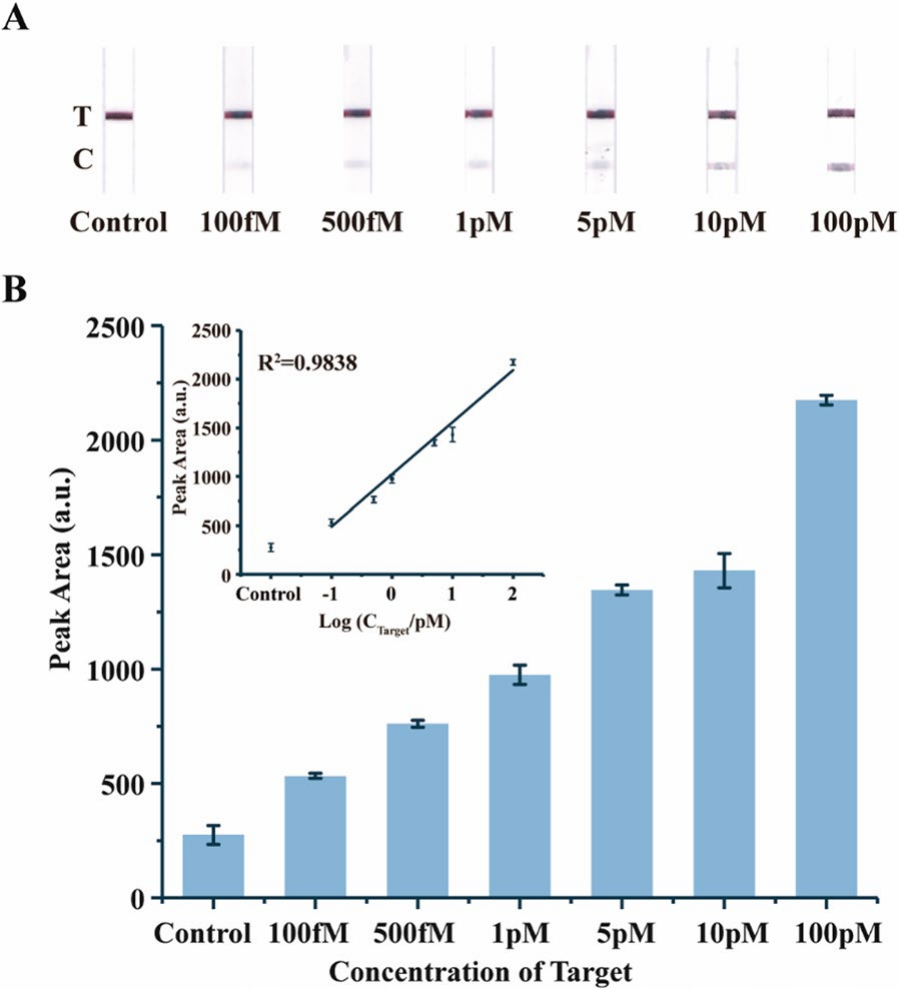
The sensitivity of the CHAGE strips for detecting target mRNA. **A** The representative photographs of strips of varying target mRNA concentrations. **B** Statistical regression analysis of the peak areas on the test zones of **A**. Error bars represent mean ± s.d., n = 3

Under optimized experimental conditions, the sensitivity of the presented method is examined using the grayscale of the test line after the two amplification steps. Different concentrations of target mRNA, from 100 fM to 100 pM, are tested (Fig. 5A), and the grayscale level and linear regression analysis are analyzed (Fig. 5B). The signal intensity of 100 fM groups is distinct from the control group and show a good linear relationship (R^2^ = 0.9838) between 100 fM and 10 pM. The overall sensitivity of the CHAGE strips at mRNA detection, as compared with lateral flow nucleic acid biosensor [18], is improved by 600-fold, and the sensitivity of simple enhanced process in strips is improved by tenfold (Additional file 1: Figure S8).

Next, we analyzed the selectivity of this method by synthesizing interference sequence as target (Additional file 1: Figure S9). The response of the interference sequence has no distinction to that of the control sample, even when its concentration (10 nM) is tenfold of the target concentration (1 nM). This indicates high selectivity and specificity of the CHAGE strips in detecting mRNA in complicated samples. This indicates high selectivity and specificity of the CHAGE strips in detecting mRNA in complicated samples. At last, we tested the stability of gold signal probes in buffer and strips (Additional file 1: Figure S10). We tested the 7 time points after the gold signal probes synthesis (1 day, 3 days, 5 days, 7 days, 10 days, and 14 days). The property of gold signal probes and the detection sensitivity of target have no obvious changes in these time points. In summary, the gold signal probes are stable at least 14 days.

### EVs GPC-1 mRNA expression level comparisons of different pancreatic tumor cells

**Fig. 6:**
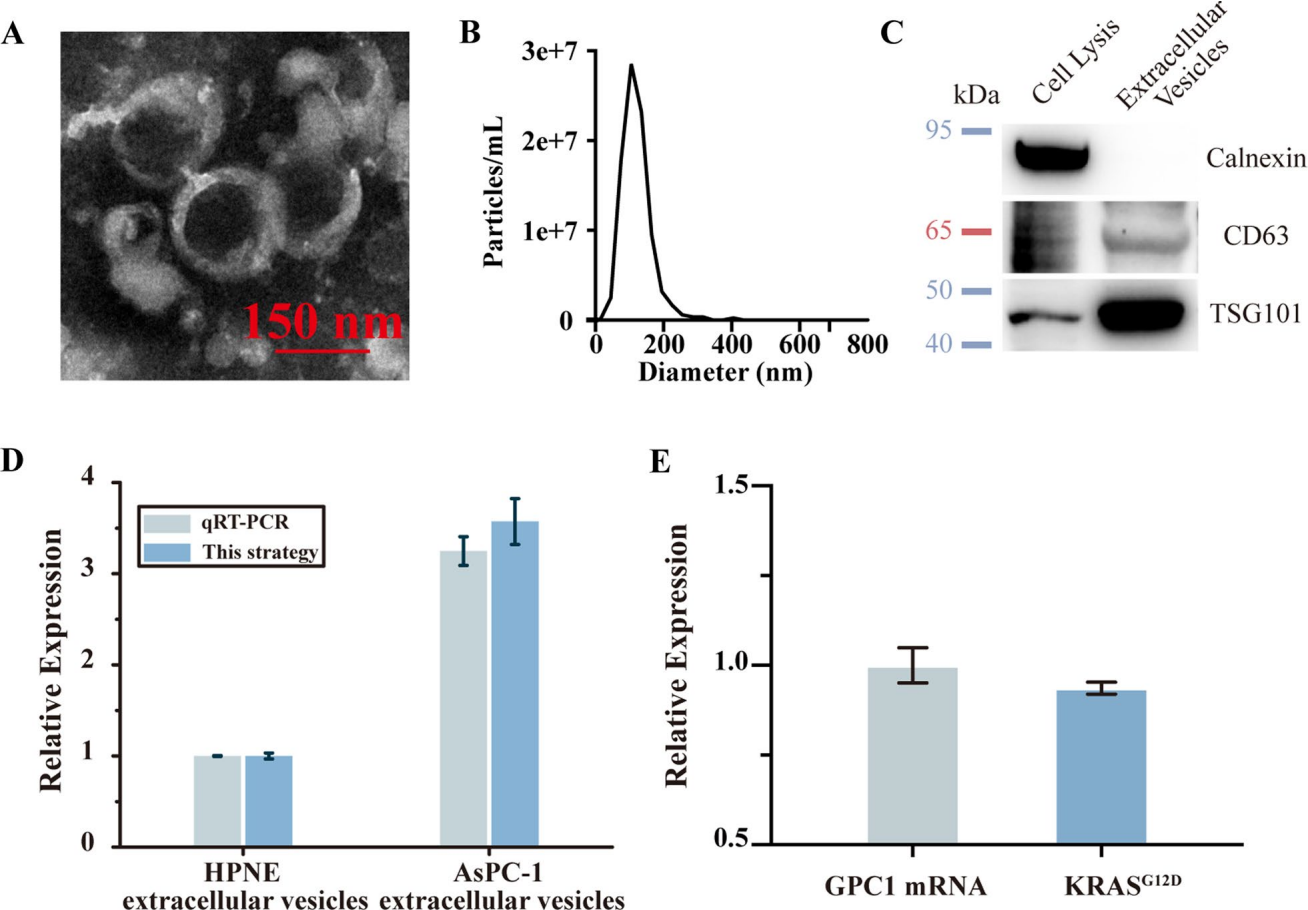
**A** The TEM of AsPC-1 extracellular vesicles. **B** The size distribution of extracellular vesicles. **C** Western blotting result of CD63, TSG101, Calnexin in AsPC-1 cell lysis and extracellular vesicles. **D** Comparison between this strategy and qRT-PCR with EVs acquired from HPNE control cells and AsPC-1 pancreatic tumor cells. **E** Comparison between GPC1 mRNA and KRAS^G12D^ mutation in AsPC-1 EVs with qRT-PCR. Error bars represent mean ± s.d., *n* = 3

To further demonstrate the practicality of this strategy with real samples, we test target mRNA GPC1 from total RNAs extracted from AsPC-1 EVs. We first isolated EVs from AsPC-1 cell lines and characterized them by TEM, western blotting, and nanoparticle tracking analysis (NTA) (Fig. 6A–C). The isolated EVs show the typical saucerlike structure in TEM and about 100 nm size distribution in NTA analysis. Positive expression of CD63 in western blotting analysis validated successful EVs isolation.

Then we applied our strategy to evaluating extracellular vesicles’ GPC1 mRNA expression between pancreatic cancer cell line (AsPC-1) and normal pancreatic cell line (HPNE). The expression of GPC1 mRNA in AsPC-1 extracellular vesicles was calculated to be 3.5-fold higher than that in HPNE extracellular vesicles and the result was cross-verified by qRT-PCR (Fig. 6D) with the same concentration of extracellular vesicles which was measured by NTA. The detection consistency between CHAGE strips and qRT-PCR revalidates the effectiveness of GPC1 mRNA portion (2034 region) as a target (Fig. 6D). This GPC1 mRNA expression tendency between normal and cancerous pancreatic cells is consistent with literature reports [5]. Given the differentiated EVs related GPC1 expression level between normal and cancer pancreatic cells, this strategy could sensitively discriminate the pancreatic cancer.

At last, we detected the expression level of EVs related KRAS^G12D^ of pancreatic cancer cell lines (Fig. 6E). KRAS^G12D^ mutation broadly exists in pancreatic cancer. Using qRT-PCR, the KRAS^G12D^ mutation obviously exists in EVs related pancreatic cancer cell line (AsPC-1) (Fig. 6E). KRAS^G12D^ is known as a great prognosis biomarker of pancreatic cancer [33]. The detection of KRAS^G12D^ mutation after GPC-1 mRNA detection could further improve the detection accuracy and forecast the effectiveness of prognosis. Therefore, the proposed CHAGE strategy presents a novel method for real sample mRNA detection for use in early cancer diagnosis and prognosis.

## Conclusions

In summary, we constructed a two-step amplification system (CHAGE strips) to detect trace-amount mRNA. A non-enzymatic amplification with hairpin sequence design provides a mild reaction condition and simple operation. Furthermore, the signal gold-enhanced strip detection method, as a portable device, is easy to operate and quickly provides results. As a result, the combination of these two amplification processes, CHA and AuNPs enhancement, enables 100 fM target mRNA detection within 2 h. The successful application of GPC1 mRNA detection in EVs indicates its potential to facilitate early detection of pancreatic cancer. This presented assay provides a common mRNA detection platform that can be adopted to detect other mRNA, including COVID-19 and virus, by designing corresponding detection probes. Given the high detection sensitivity, easy data acquirement, high specificity, and accuracy of CHAGE strips, along with a simple pre-treatment platform, it is a powerful POCT device in home-mRNA detection for disease pre-diagnosis.

## Supplementary Information

**Additional file 1: Table S1.** Sequences of all probes in experiments. **Figure S1.** Simulation of all structures produced in CHA process. **Figure S2.** The stability ofgold signal probes in high salt buffer. **FigureS3.** UV–VIS of raw gold nanoparticles and gold signal probes. **Figure S4.** UV–VIS of gold signal probes before and after amplification. **Figure S5.** The gold nanoparticles wavelength various with time in different enhancement buffer. **Figure S6.** The goldnanoparticles wavelength various with time in different enhancement buffer. **Figure S7.** The gold nanoparticles grayscale various with time in different enhancement buffer. **Figure S8.** The appearance of enhancement buffer treatment in strips. **Figure S9.** Specificity of CHAGE stripes for detection of GPC1 mRNA. **Figure S10.** The stability of gold signal probes in buffer and strips. **Table S2.** Comparison of different detection methods.

## Data Availability

All data generated or analyzed during this study are included in this published article.
