# Supplementary material for: Highly sensitive and portable mRNA detection platform for early cancer detection
Source: J Nanobiotechnology. 2021 Sep 26;19:287. doi: 10.1186/s12951-021-01039-4 (PMC8474757; doi:10.1186/s12951-021-01039-4)
Supplement: Supplementary file 1 — Additional file 1: Table S1. Sequences of all probes in experiments. Figure S1. Simulation of all structures produced in CHA process. Figure S2. The stability ofgold signal probes in high salt buffer. FigureS3. UV–VIS of raw gold nanoparticles and gold signal probes. Figure S4. UV–VIS of gold signal probes before and after amplification. Figure S5. The gold nanoparticles wavelength various with time in different enhancement buffer. Figure S6. The goldnanoparticles wavelength various with time in different enhancement buffer. Figure S7. The gold nanoparticles grayscale various with time in different enhancement buffer. Figure S8. The appearance of enhancement buffer treatment in strips. Figure S9. Specificity of CHAGE stripes for detection of GPC1 mRNA. Figure S10. The stability of gold signal probes in buffer and strips. Table S2. Comparison of different detection methods. [file 12951_2021_1039_MOESM1_ESM.docx]

**Additional file 1**

**Highly Sensitive and Portable mRNA Detection Platform for Early Cancer Detection**

Hongxia Li^a^, Antony R. Warden^a^, Wenqiong Su^a^, Jie He^a^, Xiao Zhi^a^, Kan Wang^b^, Laikuan Zhu^c,d,*^, Guangxia Shen^a,^*, Xianting Ding^a,^*

a. State Key Laboratory of Oncogenes and Related Genes, Institute for Personalized Medicine, School of Biomedical Engineering, Shanghai Jiao Tong University, Shanghai, 200030, China.

b. Institute of Nano Biomedicine and Engineering, Shanghai Engineering Research Centre for Intelligent Diagnosis and Treatment Instrument, Department of Instrument Science and Engineering, School of Electronic Information and Electrical Engineering, Shanghai Jiao Tong University, Shanghai 200240, China

c. Department of Endodontics and Operative Dentistry, Ninth People’s Hospital, Shanghai Jiao Tong University School of Medicine, Shanghai,200030, China

d. Shanghai Key Laboratory of Stomatology & Shanghai Research Institute of Stomatology; National Clinical Research Center of Stomatology, Shanghai, 200030, China

* Correspondence to

Laikuan Zhu,

Department of Endodontics and Operative Dentistry, Ninth People’s Hospital, Shanghai Jiao Tong University School of Medicine, Shanghai Key Laboratory of Stomatology & Shanghai Research Institute of Stomatology; National Clinical Research Center of Stomatology.

E-mail: [zhulk1997@163.com](mailto:zhulk1997@163.com)

Guangxia Shen, PhD, Associate professor

State Key Laboratory of Oncogenes and Related Genes, Institute for Personalized Medicine, School of Biomedical Engineering, Shanghai Jiao Tong University

E-mail: gxshen@sjtu.edu.cn

Xianting Ding, PhD, Professor

State Key Laboratory of Oncogenes and Related Genes, Institute for Personalized Medicine, School of Biomedical Engineering, Shanghai Jiao Tong University.

E-mail: dingxianting@sjtu.edu.cn

Table of Contents

- Sequences of all probes in experiments. (Table S1)
- Simulation of all structures produced in CHA process. (Figure S1)
- The stability of gold signal probes in high salt buffer. (Figure S2)
- UV-VIS of raw gold nanoparticles and gold signal probes. (Figure S3)
- UV-VIS of gold signal probes before and after amplification. (Figure S4)
- The gold nanoparticles wavelength various with time in different enhancement buffer. (Figure S5)
- The gold nanoparticles wavelength various with time in different enhancement buffer. (Figure S6)
- The gold nanoparticles grayscale various with time in different enhancement buffer. (Figure S7)
- The appearance of enhancement buffer treatment in strips. (Figure S8)
- Specificity of CHAGE stripes for detection of GPC1 mRNA. (Figure S9)
- The stability of gold signal probes in buffer and strips. (Figure S10)
- Comparison of different detection methods. (Table S2)

**Table S1.** Sequences of all probes in experiments

| **Note** | **Sequence (5’-3’)** |
| --- | --- |
| **Hairpin probe 1**  **(H1)** | GCC TGC CCC TGC TCA GAG AGA ATG TGA ACA CTC TGA GCA GGC CTT GTC ATA GA |
| **Hairpin probe 2**  **(H2)** | CAG AGT GTT CAC ATT CTC TCT GAG CAT AAG AAT GTG AAC AGA CAC CAT TT |
| **Target 2034 zone** | CTC TGA GCA GGG GCA GGC |
| **Signal probe** | (5’SH-C6) TCT ATG ACA AGG |
| **Control probe** | (5’Biotin) CCT TGT CAT AGA |
| **Test probe** | (5’Biotin) AAA TGG TGT C |
| **Interference zone** | CUC GGA GUA GGA GCG GGC |
| **miR39 forward** | GCGTCACCGGGTGTAAATC |
| **miR39 reverse** | AGTGCAGGGTCCGAGGTATT |
| **KRAS^G120D^ forward** | ACTTGTGGTAGTTGGAGCAGA |
| **KRAS^G120D^ reverse** | TTGGATCATATTCGTCCACAA |
| **2034 forward** | CTATTGCCGAAATGTGCTCAAG |
| **2034 reverse** | GATGTACCCCAGAACTTGTCG |
|  |  |

**Figure S1:**


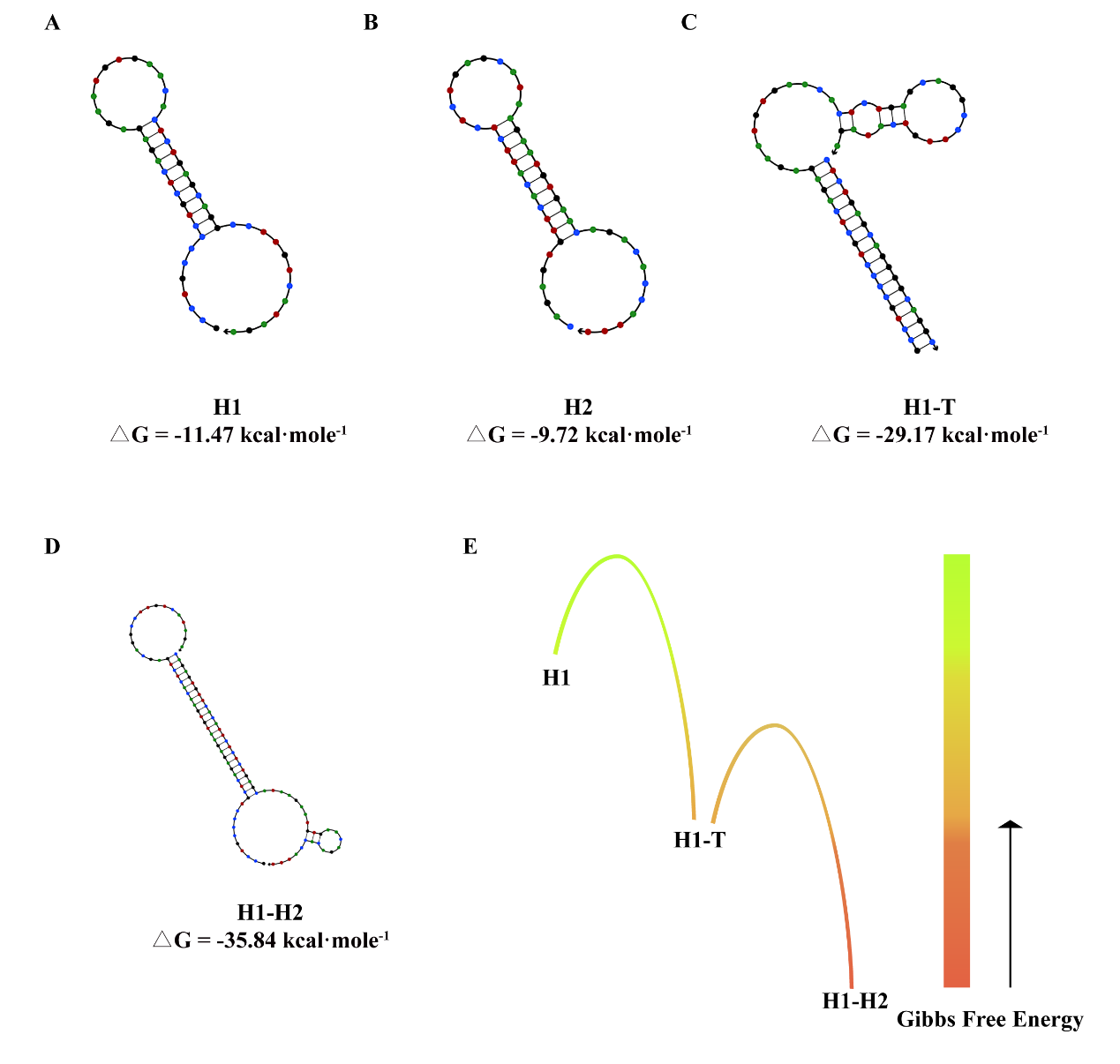


Figure S1. Simulation of all structures produced in CHA process. A. H1 (Hairpin 1) structure. B. H2 (Hairpin 2) structure. C. H1-T (H1 and Target complex) structure. D.H1-H2 (H1 and H2 complex) structure. E. Variation of Gibbs free energy in CHA process.

**Figure S2:**


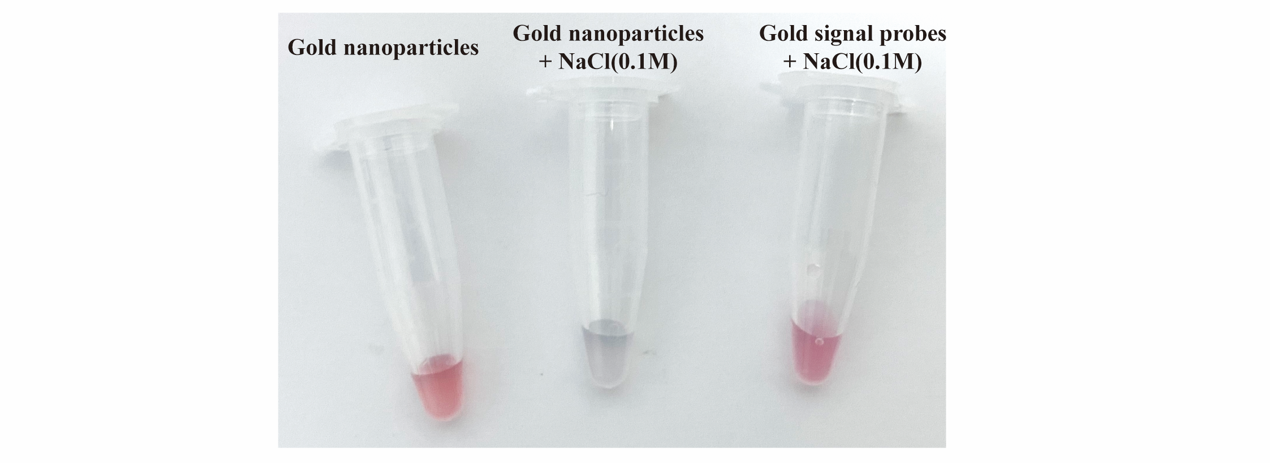


Figure S2. Images of gold nanoparticles, gold nanoparticles with 0.1M NaCl and gold signal probes with 0.1M NaCl.

**Figure S3:**


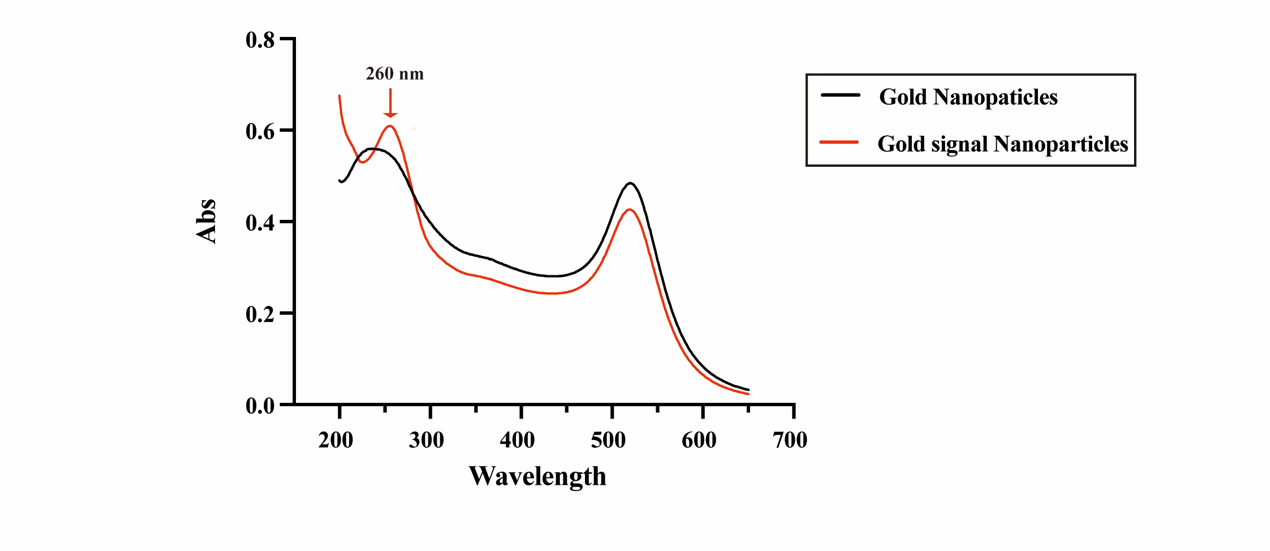


Figure S3. UV-VIS of raw gold nanoparticles and gold signal nanoparticles.

**Figure S4:**


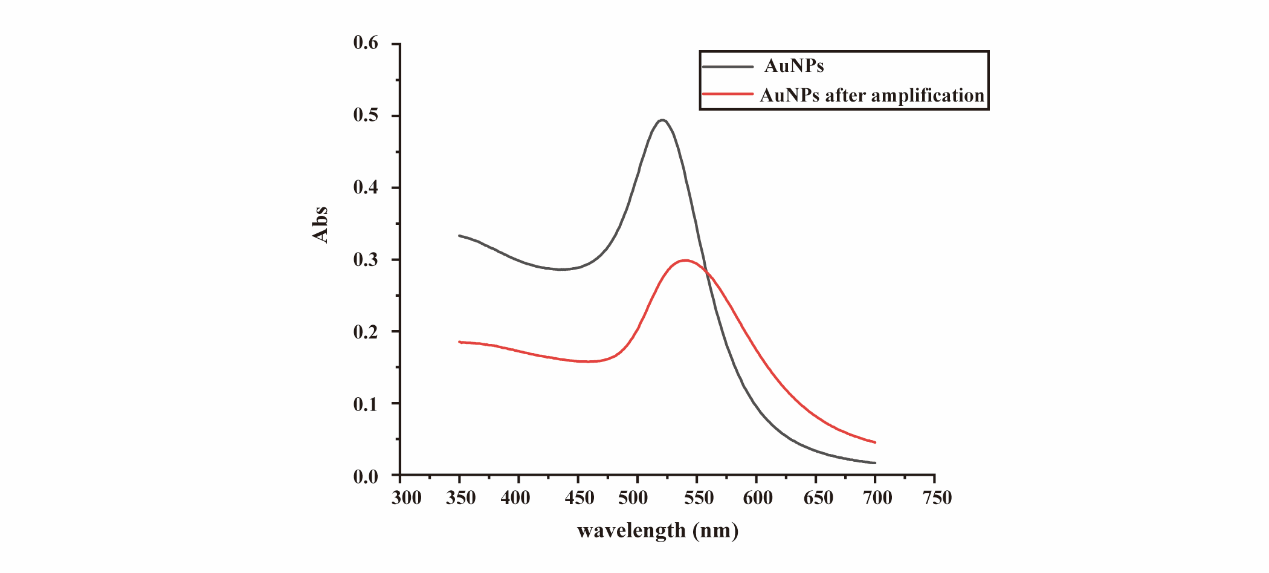


Figure S4. UV-VIS of gold signal probes before and after amplification.

**Figure S5:**


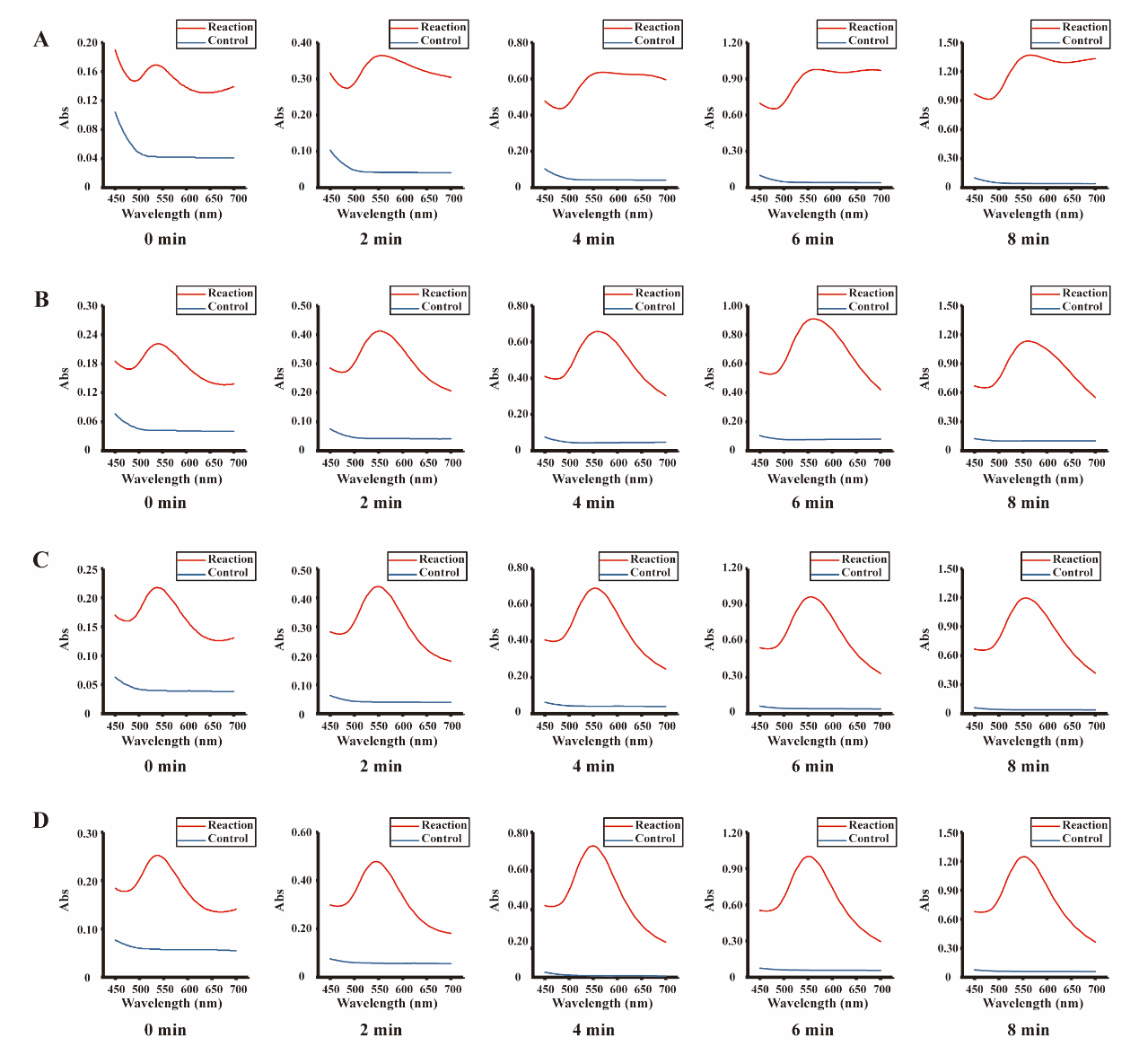


Figure S5. The gold nanoparticles wavelength various with time in different enhancement buffer. A: 1:5, B: 1:10, C: 1:15, D: 1:20. Enhancement buffer: 1% HAuCl_4_ and 10mM NH_4_OH·HCl. The ratio represents 1% HAuCl4:10mM NH_4_OH·HCl (v/v).

**Figure S6:**


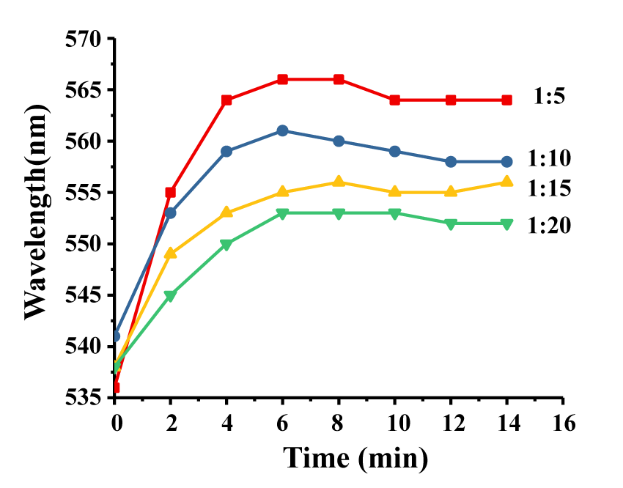


Figure S6. The gold nanoparticles wavelength various with time in different enhancement buffer. The ratio represents 1% HAuCl_4_:10 mM NH_4_OH·HCl (v/v).

**Figure S7:**


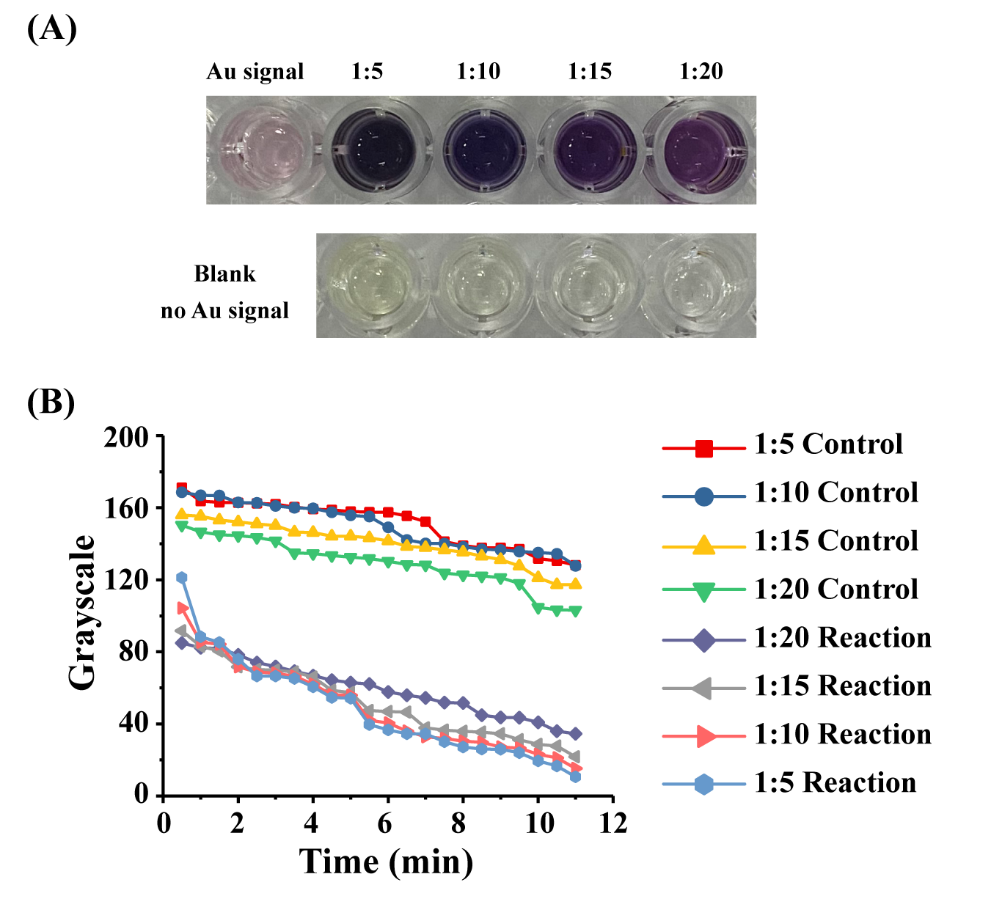


Figure S7. The gold nanoparticles grayscale various with time in different enhancement buffer. A: the photograph of terminal sample. B. the grayscale various with different time treatment. The ratio represents 1% HAuCl_4_ : 10 mM NH_4_OH·HCl (v/v). Enhancement buffer: 1% HAuCl_4_ and 10 mM NH_4_OH·HCl.

**Figure S8:**


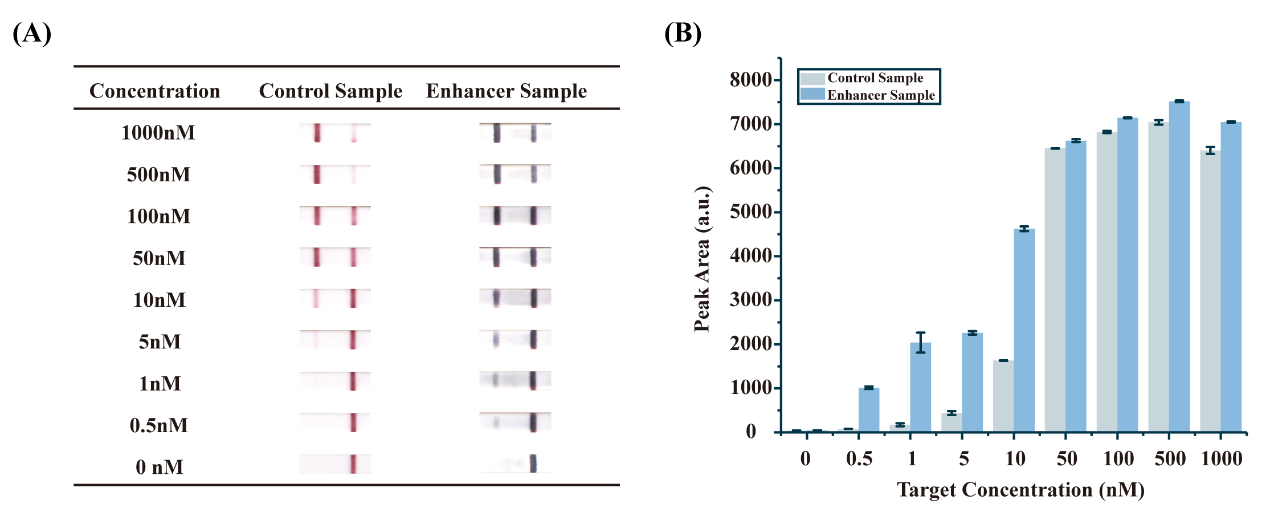


Figure S8. The appearance of enhancement buffer treatment in strips. A. Different concentration of H1-H2 complex (strips target) performance in the strips before and after enhancement buffer treatment. B. The histogram of T zones peak area on different strips of (A). The enhancement buffer: 1% HAuCl_4_:10 mM NH_4_OH·HCl =1:10 (v/v).

**Figure S9:**


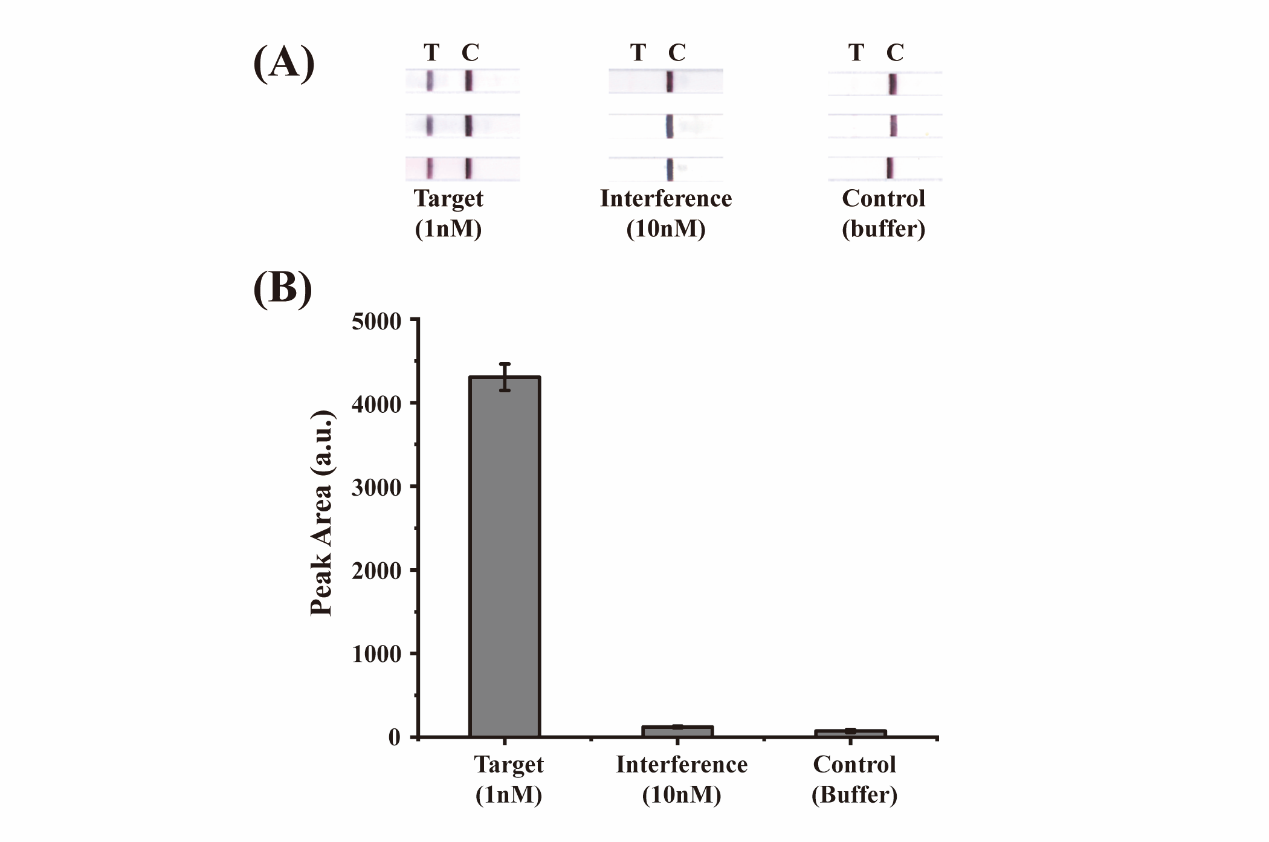


Figure S9. Specificity of CHAGE strips for detection of GPC1 mRNA. (A) The photographs of CHAGE stripes with different kinds of samples: Target (2034 portion, 1 nM), Interference (10 nM), Control (buffer). (B) Histogram of the peak areas on the test zones of (A).

**Figure S10:**


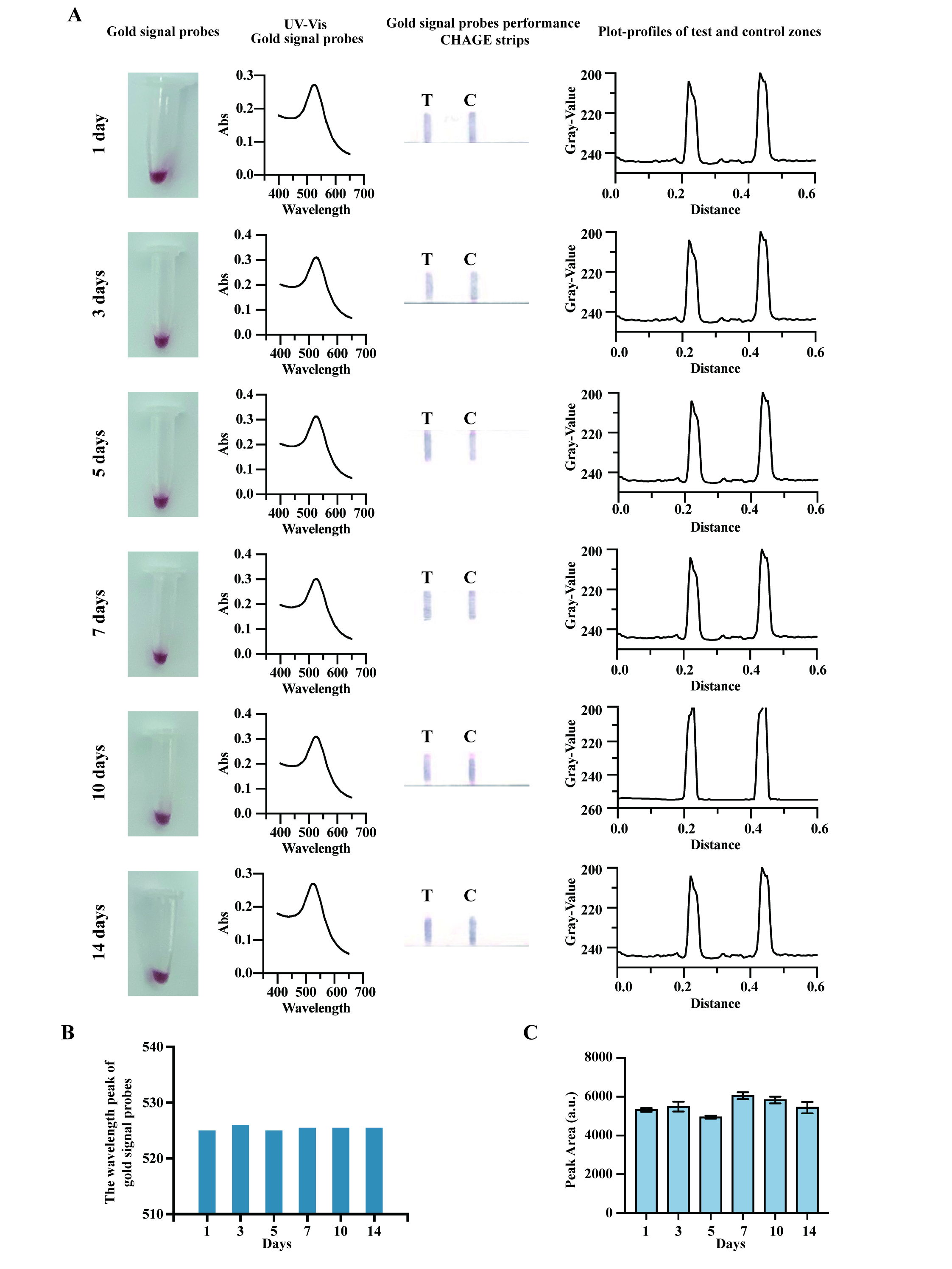


Figure S10. The stability of gold signal probes. (A) The stability evaluations from four aspects: the color of gold signal probes, the UV-Vis of signal probes, the performance in CHAGE strips and the plot profiles of the strips. (B). The wavelength peak of gold signal probes in 14 days. (C). The target detection stability within 14 days at optimized experimental conditions.

**Table S2** **Comparison of different detection methods**


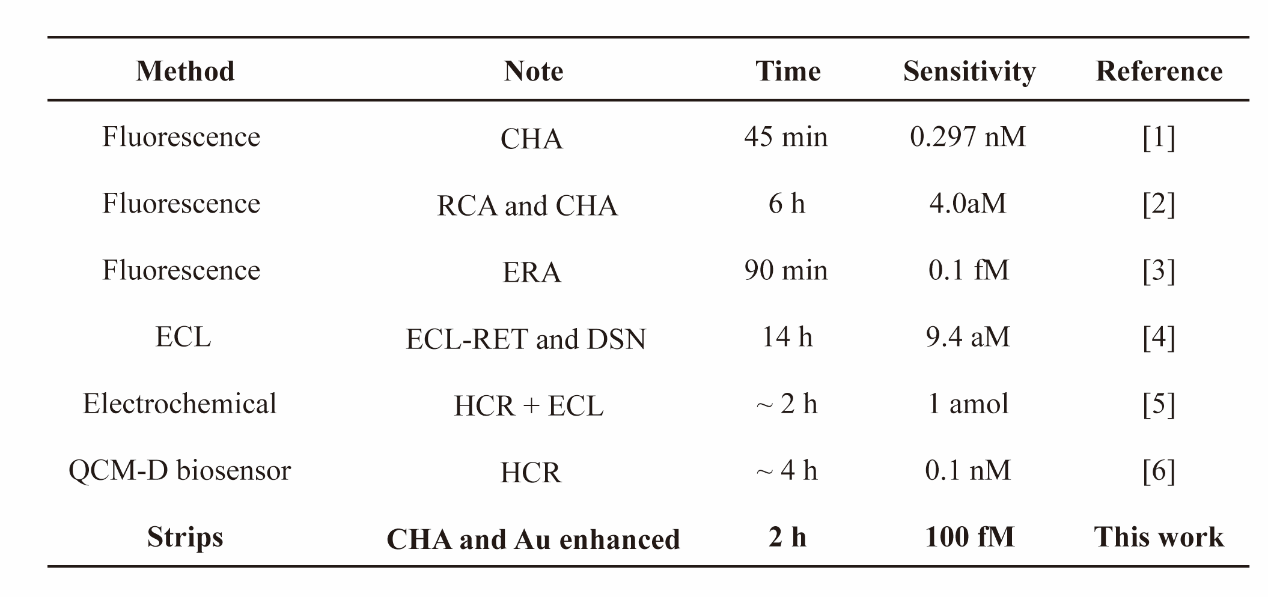


Note: CHA: catalytic hairpin assembly, RCA: rolling circle amplification, ERA: enzymatic repairing amplification, ECL: electrochemiluminescence, ECL-RET: electrochemiluminescence resonance energy transfer, DSN: duplex-specific nuclease, HCR: hybridization chain reaction.

[1] H. Kim, S. Kang, K.S. Park, H.G. Park, Enzyme-free and label-free miRNA detection based on target-triggered catalytic hairpin assembly and fluorescence enhancement of DNA-silver nanoclusters, Sensors and Actuators B: Chemical, 260 (2018) 140-145.

[2] W. Song, Q. Zhang, W. Sun, Ultrasensitive detection of nucleic acids by template enhanced hybridization followed by rolling circle amplification and catalytic hairpin assembly, Chemical communications, 51 (2015) 2392-2395.

[3] D.-M. Zhou, W.-F. Du, Q. Xi, J. Ge, J.-H. Jiang, Isothermal Nucleic Acid Amplification Strategy by Cyclic Enzymatic Repairing for Highly Sensitive MicroRNA Detection, Analytical chemistry, 86 (2014) 6763-6767.

[4] X.L. Huo, N. Zhang, H. Yang, J.J. Xu, H.Y. Chen, Electrochemiluminescence Resonance Energy Transfer System for Dual-Wavelength Ratiometric miRNA Detection, Analytical chemistry, 90 (2018) 13723-13728.

[5] Y. Liao, Y. Fu, Y. Wu, R. Huang, X. Zhou, D. Xing, Ultrasensitive Detection of MicroRNA in Tumor Cells and Tissues via Continuous Assembly of DNA Probe, Biomacromolecules, 16 (2015) 3543-3551.

[6] W. Tang, D. Wang, Y. Xu, N. Li, F. Liu, A self-assembled DNA nanostructure-amplified quartz crystal microbalance with dissipation biosensing platform for nucleic acids, Chemical communications, 48 (2012) 6678-6680.
